# Supplementary material for: Understanding incentive preferences of community health workers using discrete choice experiments: a multicountry protocol for Kenya, Uganda, Bangladesh and Haiti
Source: BMJ Open. 2019 Dec 11;9(12):e033601. doi: 10.1136/bmjopen-2019-033601 (PMC6924748; doi:10.1136/bmjopen-2019-033601)
Supplement: Author's manuscript [file bmjopen-2019-033601.draft_revisions.pdf]

BMJ Open is committed to open peer review. As part of this commitment we make the peer review history of every article we publish publicly available.

When an article is published we post the peer reviewers' comments and the authors' responses online. We also post the versions of the paper that were used during peer review. These are the versions that the peer review comments apply to.

The versions of the paper that follow are the versions that were submitted during the peer review process. They are not the versions of record or the final published versions. They should not be cited or distributed as the published version of this manuscript.

BMJ Open is an open access journal and the full, final, typeset and author-corrected version of record of the manuscript is available on our site with no access controls, subscription charges or pay-per-view fees (http://bmjopen.bmj.com).

If you have any questions on BMJ Open's open peer review process please email editorial.bmjopen@bmj.com

BMJ Open

Understanding Incentive Preferences of Community Health Workers using Discrete Choice Experiments: A multi-country protocol for Kenya, Uganda, Bangladesh and Haiti

Journal:	*BMJ Open*
Manuscript ID	bmjopen-2019-033601
Article Type:	Protocol
Date Submitted by the Author:	13-Aug-2019

Complete List of Authors:	Agarwal, Smisha ; Johns Hopkins University Bloomberg School of Public Health<br>Anaba, Udochisom; Population Council<br>Abuya, Timothy; Population Council, Reproductive Health Program<br>Kintu, Richard; Pathfinder International<br>Casseus, Alain; Zanmi Lasante<br>Hossain, Sharif; Population Council<br>Obadha, Melvin; KEMRI<br>Warren, Charlotte; Population Council

Keywords:	Community Health Workers, Community Health Systems, Incentives, Discrete Choice Experiment

SCHOLARONE™
Manuscripts

Understanding Incentive Preferences of Community Health Workers using Discrete Choice Experiments: A multi-country protocol for Kenya, Uganda, Bangladesh and Haiti

Authors:

Smisha Agarwal (smagarwa@jhsph.edu), John Hopkins Bloomberg School of Public Health, 615 N Wolfe Street, Baltimore 21205, Maryland, United States (Corresponding author)

Udochisom Anaba (uanaba@popcouncil.org), Population Council, 4301 Connecticut Ave NW # 280, Washington, DC 20008, United States

Timothy Abuya (tabuya@popcouncil.org), Population Council, Avenue 5, 3rd floor, Rose Avenue, Nairobi, Kenya

Richard Kintu (rkintu@pathfinder.org), Pathfinder International, Plot 30B, Impala Ave, Kololo, Kampala, Uganda

Alain Casseus (acasseus@pih.org), Zanmi Lasante, Santo 18A, Croix-des-Bouquets, Haiti

Sharif Hossain (sharifhossain@popcouncil.org), Population Council, House #12, Road #25/30 Gulshan-1 , Dhaka 1212, Bangladesh

Melvin Obadha (MObadha@kemri-wellcome.org), Health Economics Research Unit, KEMRI-Wellcome Trust Research Programme, Nairobi Kenya

Charlotte E Warren (cwarren@popcouncil.org), Population Council, 4301 Connecticut Ave NW # 280, Washington, DC 20008, United States

Corresponding Author:

Smisha Agarwal
Email: smagarwa@jhsph.edu
Address: Suite W5009E, 615 N Wolfe Street, Baltimore, Maryland, USA
Tel No: +1-347-768-2070

Word count: 4067

For peer review only

For peer review only

Abstract

Introduction

There is a renewed global interest in improving community health worker (CHW) programs. For CHW programs to be effective, key intervention design factors which contribute to the performance of CHWs need to be identified. The recent World Health Organization guidelines recommends the combination of financial and non-financial incentives to improve CHW performance. However, evidence gaps remain as to what package of incentives will improve their performance in different country contexts. This study aims to evaluate CHW incentive preferences to improve performance and retention which will strengthen CHW programs and help governments leverage limited resources appropriately.

Methods and analysis

A discrete choice experiment (DCE) will be conducted with CHWs in Bangladesh, Haiti, Kenya and Uganda with different levels of maturity of CHWs programs. This will be carried out in two phases. Phase I will involve preliminary qualitative research including focus group discussions and key informant interviews to develop the DCE design which will include attributes relevant to the CHW country settings. Phase II will involve a DCE survey with CHWs, presenting them with a series of choices between jobs with varying job attributes. A fractional factorial design will be used to reduce the number of choice-sets to a feasible level. Surveys will be administered in locally-appropriate languages to at least 150 CHWs from each of the cadres in each country. Random effects logit models will be used for the estimation of stated preferences given the different attributes and levels.

Ethics and dissemination

This study has been reviewed and approved by the Population Council's Institutional Review Board in New York, and appropriate ethics review boards in Kenya, Uganda, Bangladesh and Haiti. The results of the study will be disseminated through, in-country dissemination workshops, meetings with country-level stakeholders and policy working groups, print media, online blogs, and peer-reviewed journals.

For peer review only

Keywords

Community Health Workers, Community Health Systems, Incentives, Discrete Choice Experiment, Performance

For peer review only

Strengths and limitations of this study

- Assessment of multi-stakeholder perspectives to identify important job attributes will provide policy relevant and actionable choice options to be tested in the study
- Draws evidence from multiple countries with strong country-level stakeholder involvement including ministries of health, non-governmental organizations and donor agencies
- In countries with multiple cadres of CHWs, the study includes formative interviews with at least two cadres of CHWs to understand their perspectives
- As with other discreet choice experiments, a key limitation is that the job attributes presented to the CHWs are hypothetical
- The results have limited generalizability across the countries given the variation that tends to exist in the needs of CHWs across cadres, terrain and job responsibilities.

For peer review only

Introduction

The dearth of health workers, especially in areas where they are most needed, continues to be an ongoing challenge for health systems globally. The World Health Organization (WHO) forecasted a global shortage of approximately 18 million health workers by 2030.(1) Recruitment and training of community health workers (CHWs) provides tremendous potential to address this gap in human resources as well as ensure access to basic health services where the formal sector falls short.(2,3) The Alma Ata Declaration (1978), considered a critical twentieth century public health milestone, highlighted the key role of CHWs in advancing "health for all".(4) Consequently, CHW programs became the cornerstone for rapidly expanding primary healthcare services for poor populations.(5) Over the 1980's and 1990's, several pilot CHW programs reported substantial improvements in healthcare outcomes, demonstrating the potential contribution of CHWs.(6,7) However, most national level CHW programs were variably implemented and reported several challenges that limited the effectiveness of these programs.(8–10) Scaled CHW programs had high levels of attrition and overall reduced quality of care. It was argued that the poor performance of these national level programs was not due to the failure of the concept of CHWs, but the lack of ongoing training, supervision, logistical and financial support, as well as weaker linkages to the health system.(11) Often CHWs were unpaid volunteers and were not accountable to the health system. Where strategies to expand care through CHWs was employed as an alternative rather than a complement to professional care, the results were poor.(12) Several debates about selective versus comprehensive PHC approaches ensued.(10) The recent Astana Declaration (2018) has once again emphasized the critical role of primary health care in advancing Universal Health Coverage (UHC).(13) Once again, the potential contribution of CHWs to supporting UHC has come to the forefront.

As governments respond to this wave of renewed enthusiasm for CHW programs, it is critical to be responsive to the lessons from the past. Evidence from studies conducted over the past five decades have identified significant contributions as well as challenges of CHW programs. Bhutta et al. conducted a

For peer review only

systematic review of evidence to recommend several behavioral and promotional interventions which can be effectively delivered by CHWs with limited training or trained CHWs along the continuum of maternal health care. These include the promotion of reproductive health services and family planning, appropriate care seeking and antenatal care during pregnancy, and skilled care for childbirth.(14,15) Additionally, CHWs can play a critical role in several preventive and treatment interventions to support adequate maternal and child nutrition and vitamin supplementation, identification of high-risk pregnancies and childhood illnesses, and early management of preterm labor, malaria in pregnancy, infections and malnutrition in children.(14) However, it is also well-established that CHW programs can only be effective when CHWs are appropriately recruited, sufficiently trained and adequately supported. Understanding the preferences of CHWs is core to facilitating their retention and improved performance. Broadly, factors affecting the motivation and performance of CHWs can be categorized as: Intervention design factors that include but are not limited to incentives, training, supervision, monitoring & evaluation mechanisms; (16–18) and contextual factors like the community context, socio-cultural factors, work environment, economic context and health system policy and practice.(15,19,20) While there is some research on the programmatic inputs that contribute to strong CHW programs, further research on the appropriate combination of these inputs can help governments leverage limited resources appropriately. The 2018 WHO guideline on health policy and system support to optimize CHW programs identified this as a key research gaps and concluded that "evidence is not sufficiently granular to allow recommendation of specific forms of interventions, for example….which bundle of financial and non-financial incentives are most effective".(21)

In many LMICs, CHWs are often funded by government or donors.(22,23) Historically, the commitment to consistently fund comprehensive CHWs programs has wavered; often, in preference for vertical programs. Consequently, CHW programs have experienced severe challenges related to attrition and low quality.(19) While in the early years CHWs were thought of as "volunteers", the importance of integrating these health works into the formal health sector, including the provision of adequate

For peer review only

remuneration as well as other incentives has been widely researched and documented.(24–26) The 2018 WHO guideline on CHWs recommended that practicing CHWs are remunerated for their work "with a financial package commensurate with the job demands, complexity, number of hours, training and roles that they undertake". It further elaborated that incentives can include financial remuneration, including performance-based incentives, as well as non-financial incentives.(21) This was also captured in the Astana Declaration as "We will create decent work and appropriate compensation for health professionals and other health personnel working at the primary health care level………… continue to invest in the education, training, recruitment, development, motivation and retention of the PHC workforce".(13)

However, barriers to instituting these recommendations in practice remain. First, there is no agreement on strategies that would best support adequate incentivization of CHWs. Second, with respect to health worker compensation, the health sector human resource policies and practices vary considerably across countries. Third, given the optimal ratio of CHW to population, the question of affordability of scaled CHW programs to governments remains. A study of CHW program costs in 37 sub-Saharan African countries estimated that when CHWs are paid an equivalent of US$80/month by existing central government healthcare budgets, the median relative cost of a CHW program would be 27 percent of the national healthcare budget. (27)

This study aims to address this critical gap in understanding the combination of financial and non-financial incentives that will support CHWs, improve their work satisfaction and retention, and consequently influence the performance of CHW programs. The proposed study is being conducted by Frontline Health Project through Population Council, and Johns Hopkins Bloomberg School of Public Health, supported by Bill and Melinda Gates Foundation, in partnership with USAID, UNICEF and Integrating Community Health (ICH) NGO partners in Bangladesh, Uganda, Kenya and Haiti. The Frontline Health project works with global stakeholders to contribute to the objective of advancing evidence for community health. Given the active leadership and participation of donors, NGOs and governments for this study, we expect that the results will aid donors and national policymakers to

For peer review only

develop training and remuneration policies that are conducive to high performance of CHW programs, as well as maximize the effective use of limited resources.

To elicit the incentive preferences of CHWs, we propose the use of discrete choice experiments (DCEs). DCEs present respondents with two or more hypothetical choices and ask them to select their preferred alternatives. In this study, we propose to present CHWs with alternatives with different job attributes (e.g. salary, promotion, recognition etc.) that have been systematically identified through stakeholder interviews and a review of literature. Based on the selection of preferred alternatives, the influence of each attribute on the choice can be estimated mathematically. Furthermore, trade-offs respondents are willing to make can be quantified specifically the marginal willingness to accept monetary compensation. DCEs are based on Random Utility Theory (RUT), which suggests that the benefit or value (utility) that an individual derives from item A compared to item B is a function of the frequency with which (s)he chooses item A or item B, when given repeated choices.(28) RUT generally suggests that individuals are rational decision makers aiming to solve an optimization problem. They assign perceived utility to each alternative and will therefore choose the one that derives them the highest utility among a set of mutually exclusive alternatives.(29–31) These utilities are contingent on the attributes of the alternative and the characteristics of the decision maker.

This approach has been used in comparable studies involving CHWs in India and healthcare workers in LMICs.(32,33) Prior studies have used DCE to model different policy interventions on the recruitment of nurses to rural areas in Kenya, South Africa and Thailand (34). In Mozambique, DCE was used to elicit the job preferences of non-physician health professionals (35). In Tanzania and rural Vietnam, DCE has been used to understand how to make jobs in rural areas more attractive to medical students and doctors respectively.(36,37)

For peer review only

Methods and Analysis

We will use a DCE to examine the job attributes that influence the overall satisfaction and motivation of CHWs in Bangladesh, Haiti, Kenya and Uganda. For the study, we focus on a set of factors that are amenable to national policy and programmatic changes. The study will be conducted in two phases. In the first phase, we aim to identify a set of attributes that are realistic, pragmatic and meaningful to CHWs and policymakers, and in the second phase, we will prioritize critical attributes associated with job satisfaction from the CHW's point of view. The study design considers the varying context across the four countries, as well as the multiple cadres and roles of the CHWs in each country.

Study setting and participants

Bangladesh has 8 administrative divisions, 64 districts, and 491 sub-districts (Upazilas). Each rural area within an upazila is divided into union parishads and mouzas, which are further divided into villages. An urban area in an upazila is divided into wards, and mohallas within each ward.(38) The smallest units within rural areas are the villages and CHWs operate at this level. Bangladesh has several cadres of health workers operating at the community-level. Health Assistants (HA), Family Welfare Assistants (FWA) and Community Health Care Providers (CHCP) are commonly referred to as CHWs in the public sector, and they work within the purview of primary health care centers known as Union Health and Family Welfare Centers. Currently, the government is also recruiting multipurpose volunteers and prepaid volunteers in areas where vacancies and workload is higher. In addition, there are CHWs in the non-governmental systems with various names. For example, BRACs CHWS include the *Shasthya Shebikas*, which has been one of the largest cadres of CHWs in Bangladesh. However, currently the scale and function of the *Shasthya Shebikas* is being revised owing to the high attrition rate of these workers. (39)

Based on discussions with the Directorate General of Family Planning (DGFP) and Directorate General of Health Services (DGHS), the focus of the study will be on the two government-supported CHW cadres-FWAs (supported by the DGFP) and HAs (supported by the DGHS). The study will be conducted in the four Divisions of Sylhet, Rajshahi and Khulna, as well as in Chittagong Division's Cox's Bazaar area.

For peer review only

The Upazilas within each Division will be identified through further discussions with the DGFP and DGHS.

Haiti is divided into 10 geo-political regions and sub-divided into 42 states (arrondisements).(40) Each arrondisement is further divided into 142 communes in total which comprises 571 communal sections. The health system is overseen at the national level by the Ministry of Public Health and Population (MSPP) which provides policies and standards for service delivery at all levels (including at community level). The District Health Unit, supported by the Health Department Directorate, coordinates, supervises and monitors care delivery at community level.(41) The primary community health providers are the "agents de santé communautaire polyvalent" or ASCP which have been working informally in Haiti for years, but were more formally established by the MSPP in 2015 under one title and clear job description.(41)

This study will take place in three communes located in two arrondisements - one in Artibonite region and the other in Centre region. In Centre region, the study will occur in Mirebalais commune, Mirebalais arrondisement. In Artibonite region, the study will occur in Petite Riviere de L'Artibonite (PRA) commune and Verettes commune. Study sites and communal sections were selected in collaboration with Zanmi Lasante who have a strong collaboration and presence with the ASCPs in Haiti as well as the MSPP.

Kenya is made up of 47 counties with a population projection of more than 40 million. The healthcare system in Kenya is based on the 2010 Kenyan Constitution (42) which operates a decentralized system of government with the National government and 47 county governments. Kenya has a hierarchical four-tier healthcare system at the community, primary care, secondary referral and tertiary referral levels with Community Health Volunteers (CHVs) operating at the community level. The community services comprise all community-based demand creation activities and health services organized around a

For peer review only

comprehensive community strategy defined for the health sector. The primary care services comprise all dispensaries, health centres, and maternity and nursing homes in both public and private sectors. The county referral services include hospitals operating in and managed by a county both public and private which form the county referral system.(43) Every 1,000 household (approx. 5,000 people) are covered by one Community Health Unit (CHU), comprising of a Community Health Committee (CHC) and 5 of Community Health Extension Workers (CHEWs). CHEWs supervise Community Health Volunteers (CHVs) who are embedded within community health units (CHUs). CHVs are supposed to be supervised at the community level by community health committees (CHCs).

In Kenya, the study will take place in Bungoma, Kilifi counties and CHVs will be the primary survey respondent for phase II of the study. Kilifi is a coastal county with mixed rural urban populations, and Bungoma is an agrarian county in Western Kenya. These counties represent a set of diverse vulnerabilities within which CHWs operate. Within each of these counties, two sub-counties will be selected in consultation with the county health management teams.

In Uganda, the Health Sector Development Plan highlights the critical shortage of health workers, with a ratio of 1.55 health workers per 1000, compared to the WHO recommendations of 2.28 per 1000. Since 2001, in Uganda, Village Health Teams (VHT) were established to support community health through health promotion and education activities, mobilization of communities for utilization of health services, and treatment of illnesses at the community-level. In 2014, based on a comprehensive VHT assessment, it was recommended that the VHT strategy is overhauled, along with consideration of a standardized approach for incentives for all VHTs.(44) In response to these findings, the Ministry of Health is in the process of discussing a new Community Health Extension Worker (CHEW) strategy in the recent years. Under this new strategy, CHEWs will be adequately trained in promotive and basic curative health services, and their role will encompass supervising the VHTs. However, at the time of conducting the study, the CHEW policy is pending cabinet approval. Hence, for this study, CHWs, VHTs, policymakers

For peer review only - http://bmjopen.bmj.com/site/about/guidelines.xhtml

and other CHW supervisors will be interviewed during phase 1. Based on detailed discussions with the MoH and other stakeholders, the study will be conducted sub-counties acorss eight counties — Lira, Mayuge, Wakiso, Ntungamo, Kabale, Arua, Kabarole and Nakapiripiriti — to facilitate greater representation of the cadres of CHWs across the country. These counties were selected based on considerations for the types of terrain and tribes where CHWs serve.

Phase 1: Sampling and approach to inform the development of attributes and levels

In the first phase, an initial set of policy-relevant, realistic and actionable attributes will be identified based on a review of secondary literature, as well as primary research involving focus group discussions (FGDs) with a sample of CHWs, their supervisors, and national-level stakeholders involved in making decisions about CHW programs in the country. The attributes and attribute levels will be established separately for each country, based on information collected through this formative phase. Discussion topics will include expected remuneration, working conditions, availability of supplies and equipment, amount of training, supervision, job role and expected support from the health system and community. The FGD will use a nominal group technique where the facilitator will ask the participants to think about aspects of the job that are most important to CHWs as well as actionable by policymakers. Participants will silently generate a list of attributes, and then be asked to state a single attribute to the group.(45) This will be recorded on a board. This will continue until saturation of attributes is reached. Following this, a discussion to identify the levels of the attributes will be initiated. Participants will be asked to rank order the identified attributes and their levels privately based on their preferences. While there are no restrictions on the number of attributes that can be included in a DCE, in practice, fewer than 10 attributes should be selected to ensure that respondents can consider all attribute.(46) Definitions of each attribute will be defined, appropriate to the setting, and any overlap or correlation between the attributes will be avoided. Base levels for each attribute will be established to reflect the prevailing working conditions in the country for government-supported CHWs. Additional levels will be then determined to represent a

For peer review only

reasonable improvement from the base level. Levels will be chosen to reflect a range of situations that CHWs might realistically expect to experience.

A total of ~4 FGDs with 5-7 national and county/subcounty level policy makers, 4-6 FGDs with CHW supervisors and ~6-8 FGDs with CHWs (each cadre) will be conducted in each country. Additionally, for policy-makers who cannot be convened in a group setting, the same set of interview questions will be asked to them as key informants in an in-depth interview format. All participants must be aged 18 years or and with the ability to provide consent. There are no eligibility criteria related to gender or marital status. Policymakers will be identified by the study team and other relevant partners and CHWs and their supervisors will be recruited through community health units/primary health care facilities in select counties/districts. To ensure that the study takes a ground-up approach, the first set of interviews will be conducted with CHWs and supervisors. The study team will review the results of these interviews to then align the discussion with the policymakers and other stakeholders to reflect the priorities of the CHWs.

Prioritizing attributes and determining choice sets

Attributes and levels identified in phase 1 across all the interviews will be finalized by the study team based on the discussion transcripts. To ensure that the final set of attributes and levels represent a pragmatic and desirable set, they will be further vetted with external stakeholders such as ministry representatives and non-governmental organization leaders working in the CHW space. Efforts will be made to have clear definitions of each attributes and avoid conceptual overlaps across attributes and levels. It is recommended that DCEs should avoid having respondents consider more than 18 choice sets, at which point fatigue commonly sets in.(47) To reduce the number of choices to a feasible number for testing, we will use a fractional factorial design which is orthogonal (that each attribute is statistically independent of each other) and balanced (each level of the attribute occurs equally often), using Sawtooth software Inc. (Sequim, WA, USA).(48) To generate an efficient design, we will ensure minimal overlap

For peer review only

between attribute level within one task. The experiment will be unlabeled consisting of two job alternatives with an opt-out option.

The survey accompanying the DCE will include questions about respondent demographics, content and years of training as a CHW, socio-economic status, and current level and quality of supervision. The order of the choice sets will be randomized to avoid positional bias. In each questionnaire, one choice option will be included that one job is superior to the other on all characteristics so that internal consistency and rationality of the responses can be considered during analysis.(47) Finally, the questionnaires will be pre-tested with a sub-set of 20-30 respondents and changes made to the content and wording of the questions to account for any conceptual overlap and lack of clarity.

Phase 2: Sampling and approach for the DCE

In each country, the questionnaire will be translated into local language/s. The survey will be administered face-to-face to the CHWs by trained data collectors on tablets using the Open Data Kit (ODK) software (49). First, the purpose of the study will be explained. The data collectors will explain the meaning of each attribute to the CHWs to support comprehension of the choice tasks. Each CHW will consider a set of job descriptions and will be asked to choose a job that would best support them given their circumstances. They will also have to option to choose neither of the jobs presented to them.

The minimum sample size needed to detect differences in the preferences is dependent on the following factors; level of significance, statistical power, method of analyzing the DCE data (e.g. conditional logit, mixed multinomial logit), experimental design, and initial beliefs about parameter values.(50) However, in practice, sample size calculation in DCE studies mostly rely on rule-of-thumb estimates.(45) For estimation of a DCE model, Lancsar & Louviere (51) suggested that if respondents receive the same design, one rarely requires more than a sample size of 20 respondents. Other studies have shown that sample sizes of 40–100 respondents or up to 300 respondents may be adequate for reliable statistical

For peer review only

analysis.(50) The sample size calculation or this study is guided by the rule-of-thumb approach proposed by Johnson and Orme, as well as expected number of choice sets and pragmatic considerations around time and budget to conduct the study across the four countries.(52,53) We estimate recruiting a minimum of 150 CHW per cadre per country to be a sufficient number of respondents for the desired analysis. A total of approximately 900 respondents across the four countries will be recruited for Phase 2. Based on the total sample size, an equal number of CHWs will be recruited per geographic area in discussion with the county/district health management teams to get broad representation of the types of CHWs.

Public and Patient involvement

The first phase of the study is structured to formally gather feedback from the broader community that supports CHWs, including national and international stakeholders and donors, other health officials, CHW supervisors and CHWs themselves. Opinions and experiences of these stakeholders are critical to shaping the questionnaire design and approach for the second phase of the study. Health officials at the facilities will be involved in the identification and recruitment of the CHWs in Phase 2 of the study. Study results will be disseminated through in-country workshops where health officials involved during the design and recruitment phase will be invited to participate.

Analysis Plan

Based on all the responses, for each country, the significant levels of the selected job attributes will be analyzed using a logistic regression model in Stata 16 software (54). The regression model for this analysis will specify the likelihood of choosing one job over another given the differences in the levels of the attributes. Demographic and training characteristics will be accounted for in the analysis. Where feasible, the marginal willingness to accept estimates will be computed to understand the amount of money CHWs are willing to accept to compensate for improvement of a job attribute. To relax some of the assumptions of the logistic model around the independence of the alternatives, we will also conduct the analysis using a mixed multinomial logit model, which assumes that all the parameters are randomly distributed. We may further explore opportunities for regional or multi-country analysis, if appropriate.

For peer review only

Limitations of the study in this context

Intrinsic and extrinsic factors influencing CHW incentive preferences are diverse and real-life decisions change over time, therefore the relative importance of job preferences may vary with time and this needs to be considered when designing CHW programs and policy interventions. We also recognize that CHW preferences can be different based on their organizational affiliations, and prior experiences working as CHWs. While the study aims to capture a sample of CHWs across few areas in each country, the sample is not nationally representative of the preferences of all CHWs. We recognize that a comprehensive job package for CHWs may comprise of many more factors than can be adequate captured by this study design. Finally, the results of this study will constitute stated preferences, reflecting responses to hypothetical scenarios. Decision makers will need to consider information from DCEs on CHW as part of the inputs to improved CHW performance and retention while making policies.

For peer review only

Ethics and dissemination

This study has been reviewed and approved by the Population Council's Institutional Review Board in New York, the AMREF-Health Africa Ethics and Scientific Review Committee in Kenya, Zanmi Lasante's IRB (Haiti) and the Higher Degrees Research Ethics Committee at Makerere University College of Health Sciences in Uganda, and the Bangladesh Medical Research Council (BMRC). Dissemination activities will include sharing of findings through, policy briefs, workshops, online blogs in community fora, and peer-reviewed journal articles. The research approach is inherently designed around the need for research uptake by policy-makers. Involvement in the research from the beginning is the over-arching strategy for promoting research uptake. To ensure there is significant demand for the final study findings, we will debrief policy makers and other health system stakeholders at pivotal points in the initial stages as well as at the end of the study. We will share non-technical summaries of research results with all the partners for dissemination including the study's key partners in the Ministry of Health. If there is enough interest, appropriate dissemination will be conducted with policymakers in half-day workshops, and to facilitate translation of the research findings in national CHW agenda.

For peer review only

References

1. Darzi A, Evans T. The global shortage of health workers-an opportunity to transform care. Lancet (London, England) [Internet]. 2016 Nov 26 [cited 2018 Aug 26];388(10060):2576–7. Available from: http://www.ncbi.nlm.nih.gov/pubmed/27894651

2. Perry HB, Zulliger R, Rogers MM. Community Health Workers in Low-, Middle-, and High-Income Countries: An Overview of Their History, Recent Evolution, and Current Effectiveness. Annu Rev Public Heal. 2014;35:399–421.

3. O'Donovan J, O'Donovan C, Kuhn I, Sachs SE, Winters N. Ongoing training of community health workers in low-income and middle-income countries: a systematic scoping review of the literature. BMJ Open [Internet]. 2018 Apr 1;8(4). Available from: http://bmjopen.bmj.com/content/8/4/e021467.abstract

4. World Health Organisation. Declaration of Alma-Ata: International Conference on Primary Health Care, Alma-Ata, USSR, 6-12 [Internet]. 1978 [cited 2019 Aug 8]. Available from: https://www.who.int/publications/almaata_declaration_en.pdf

5. Christopher JB, Le May A, Lewin S, Ross D a. Thirty years after Alma-Ata: a systematic review of the impact of community health workers delivering curative interventions against malaria, pneumonia and diarrhoea on child mortality and morbidity in sub-Saharan Africa. Hum Resour Health. 2011;9(1):27.

6. Bang AT, Bang RA, Baitule SB, Reddy MH, Deshmukh MD. Effect of home-based neonatal care and management of sepsis on neonatal mortality: field trial in rural India. Lancet (London, England). 1999 Dec;354(9194):1955–61.

7. Chowdhury AM, Chowdhury S, Islam MN, Islam A, Vaughan JP. Control of tuberculosis by community health workers in Bangladesh. Lancet (London, England). 1997 Jul;350(9072):169–72.

8. Gilson L, Walt G, Heggenhougen K, Owuor-Omondi L, Perera M, Ross D, et al. National community health worker programs: How can they be strengthened? J Public Health Policy. 1989;10(4):518–32.

9. Frankel S. The community health worker: effective programmes for developing countries. Oxford University Press; 1992.

10. Rosato M, Laverack G, Grabman LH, Tripathy P, Nair N, Mwansambo C. Community participation: lessons for maternal, newborn, and child health. Lancet [Internet]. 2008;372. Available from: https://doi.org/10.1016/S0140-6736(08)61406-3

11. Lawn JE, Lawn JE, Rohde J, Rohde J, Rifk S, Rifk S, et al. Series Alma-Ata: Rebirth and Revision 1 Alma-Ata 30 years on: revolutionary, relevant, and time to revitalise. Lancet, The. 1978;917–27.

12. World Health Organization. Primary health Care - Now more than ever. Vol. 26, The World Health Report. 2008.

13. World Health Organisation & UNICEF. Declaration of Astana: Global Conference on Primary Healtcare [Internet]. 2018 [cited 2019 Aug 8]. Available from: https://www.who.int/docs/default-source/primary-health/declaration/gcphc-declaration.pdf%0D

14. Bhutta ZA, Ali S, Cousens S, Ali TM, Haider BA, Rizvi A, et al. Interventions to address

maternal, newborn, and child survival: what difference can integrated primary health care strategies make? Lancet. 2008;372(9642):972–89.

15. Haines A, Sanders D, Lehmann U, Rowe AK, Lawn JE, Jan S. Achieving child survival goals: potential contribution of community health workers. Lancet. 2007;369.

16. Nsabagasani X, Jesca Nsungwa S, Kallander K, Peterson S, Pariyo G, Tomson G. Home-based management of fever in rural Uganda: community perceptions and provider opinions. Malar J. 2007;6.

17. Puett C, Coates J, Alderman H, Sadler K. Quality of care for severe acute malnutrition delivered by community health workers in southern Bangladesh. Matern Child Nutr. 2013;9.

18. Takasugi T, Lee ACK. Why do community health care workers volunteer: a qualitative study in Kenya. Public Health [Internet]. 2012;126. Available from: https://doi.org/10.1016/j.puhe.2012.06.005

19. Kok MC, Kane SS, Tulloch O, Ormel H, Theobald S, Dieleman M, et al. How does context influence performance of community health workers in low- and middle-income countries? Evidence from the literature. Heal Res Policy Syst [Internet]. 2015 Mar;13(1):13. Available from: https://doi.org/10.1186/s12961-015-0001-3

20. Viswanathan K, Hansen PM, Hafizur Rahman M, Steinhardt L, Edward A, Arwal SH. Can community health workers increase coverage of reproductive health services? J Epidemiol Community Heal. 2012;66.

21. WHO. Guideline on health policy and system support to optimize community health worker programmes [Internet]. 2018 [cited 2019 Mar 20]. p. 1–116. Available from: https://apps.who.int/iris/bitstream/handle/10665/275474/9789241550369-eng.pdf?ua=1

22. Kok MC, Ormel H, Broerse JEW, Kane S, Namakhoma I, Otiso L, et al. Optimising the benefits of community health workers' unique position between communities and the health sector: A comparative analysis of factors shaping relationships in four countries. Glob Public Health [Internet]. 2017;12(11):1404–32. Available from: https://doi.org/10.1080/17441692.2016.1174722

23. Phiri SC, Prust ML, Chibawe CP, Misapa R, van den Broek JW, Wilmink N. An exploration of facilitators and challenges in the scale-up of a national, public sector community health worker cadre in Zambia: A qualitative study. Hum Resour Health [Internet]. 2017 Jun;15(1):40. Available from: https://doi.org/10.1186/s12960-017-0214-3

24. Karabi B, Leban K, Winch P, Tien M. Community Health Worker Incentives and Disincentives: How They Affect Motivation, Retention, and Sustainability [Internet]. Basic Support for Institutionalizing Child Survival Project (BASICS II) for the United States Agency for International Development; 2001. Available from: web.worldbank.org/archive/website01539/WEB/IMAGES/PNACQ722.PDF%0D

25. Singh D, Negin J, Otim M, Orach CG, Cumming R. The effect of payment and incentives on motivation and focus of community health workers: five case studies from low- and middle-income countries. Hum Resour Health [Internet]. 2015;13(1):58. Available from: https://doi.org/10.1186/s12960-015-0051-1

26. Scott K, Beckham SW, Gross M, Pariyo G, Rao KD, Cometto G, et al. What do we know about community-based health worker programs? A systematic review of existing reviews on community health workers. Hum Resour Health [Internet]. 2018 Aug;16(1):39. Available from: https://doi.org/10.1186/s12960-018-0304-x

For peer review only

27. Taylor C, Griffiths F, Lilford R. Affordability of comprehensive community health worker programmes in rural sub-Saharan Africa. BMJ Glob Heal [Internet]. 2017 Sep 25;2(3):e000391. Available from: http://www.ncbi.nlm.nih.gov/pmc/articles/PMC5623259/

28. Robyn PJ, Shroff Z, Zang OR, Kingue S, Djienouassi S, Kouontchou C, et al. Addressing health workforce distribution concerns: a discrete choice experiment to develop rural retention strategies in Cameroon. Int J Heal Policy Manag [Internet]. 2015;4(3):169–80. Available from: http://ijhpm.com/article_2956_616.html

29. Daniel McFadden. Conditional logit analysis of qualitative choice behavior. In: Frontiers in Econometrics [Internet]. P.(ed). New York, NY: Academic Press; 1973. Available from: https://eml.berkeley.edu/reprints/mcfadden/zarembka.pdf

30. Amaya-Amaya M, Gerard K, Ryan M. Discrete choice experiments in a nutshell. Ryan M, Gerard K, Amaya-Amaya M, editors. Dordrecht: Springer; 2008.

31. Hensher DA, Rose JM, Greene WH. Applied Choice Analysis [Internet]. 2nd ed. Cambridge: Cambridge University Press; 2015. Available from: https://www.cambridge.org/core/books/applied-choice-analysis/D409294010B38CBA138AB110B26AC6B7

32. Shiratori S, Agyekum EO, Shibanuma A, Oduro A, Okawa S, Enuameh Y, et al. Motivation and incentive preferences of community health officers in Ghana: an economic behavioral experiment approach. Hum Resour Health [Internet]. 2016;14(1):53. Available from: https://doi.org/10.1186/s12960-016-0148-1

33. Abdel-All M, Angell B, Jan S, Howell M, Howard K, Abimbola S, et al. What do community health workers want? Findings of a discrete choice experiment among Accredited Social Health Activists (ASHAs) in India. BMJ Glob Heal [Internet]. 2019;4(3). Available from: https://gh.bmj.com/content/4/3/e001509

34. Blaauw D, Erasmus E, Pagaiya N, Tangcharoensathein V, Mullei K, Mudhune S, et al. Policy interventions that attract nurses to rural areas: a multicountry discrete choice experiment. Bull World Health Organ. 2010;88:350–6.

35. Honda A, Vio F. Incentives for non-physician health professionals to work in the rural and remote areas of Mozambique---a discrete choice experiment for eliciting job preferences. Hum Resour Health [Internet]. 2015 Apr;13(1):23. Available from: https://doi.org/10.1186/s12960-015-0015-5

36. Kolstad J. How to make rural jobs more attractive to health workers. Findings from a discrete choice experiment in Tanzania. Health Econ [Internet]. 2010;20. Available from: https://doi.org/10.1002/hec.1581

37. Vujicic M, Alfano M, Shengeliac B, Witter S. Attracting Doctors and Medical Students to Rural Vietnam: Insights from a Discrete Choice Experiment. Washington, D.C: World Bank; 2010.

38. Ahmed S, Alam B, Anwar I, Begum T, Huque R, Khan J, et al. Bangladesh Health System Review [Internet]. Manila; 2015. Available from: http://www.searo.who.int/entity/asia_pacific_observatory/publications/hits/hit_bangladesh/en/

39. Ahmed SM, Evans TG, Standing H, Mahmud S. Harnessing pluralism for better health in Bangladesh. Lancet [Internet]. 2013;382(9906):1746–55. Available from: http://www.sciencedirect.com/science/article/pii/S0140673613621479

40. World Bank. Country Profile: Haiti [Internet]. 2018 [cited 2018 Oct 10]. Available from: http://databank.worldbank.org/data/views/reports/reportwidget.aspx?Report_Name=CountryProfil

For peer review only

e&Id=b450fd57&tbar=y&dd=y&inf=n&zm=n&country=HTI

41. Devlin Kristen, Kimberly Farnham Egan TP-R. COMMUNITY HEALTH SYSTEMS CATALOG COUNTRY PROFILE: HAITI [Internet]. Arlington, VA; 2016 [cited 2019 Aug 8]. Available from: https://www.advancingpartners.org/sites/default/files/catalog/profiles/apc_haiti_chs_catalog_profile_0_0.pdf

42. Republic of Kenya. Constitution of Kenya [Internet]. 2010 [cited 2018 Oct 9]. Available from: www.kenyalaw.org

43. Republic of Kenya Ministry of Health. Strategy for Community Health 2014-2019: Transforming health: Accelerating the attainment of health goals [Internet]. 2014. Available from: http://guidelines.health.go.ke:8000/media/STRATEGY_FOR_COMMUNITY_HEALTH_2014-2019.pdf

44. Republic of Uganda Ministry of Health. National Village Health Teams (VHT) assessment in Uganda [Internet]. 2015 [cited 2019 Aug 12]. Available from: https://www.pathfinder.org/wp-content/uploads/2016/09/VHT-Report-with-District-Analyses-1.pdf

45. Botha W, Donnolley N, Shanahan M, Chambers GM. Assessment of the societal and individual preferences for fertility treatment in Australia: study protocol for stated preference discrete choice experiments. BMJ Open [Internet]. 2018;8(2). Available from: https://bmjopen.bmj.com/content/8/2/e020509

46. DeShazo J, Fermo G. Designing choice sets for stated preference methods: the effects of complexity on choice consistency. J Env Econ Manag [Internet]. 2002;44. Available from: https://doi.org/10.1006/jeem.2001.1199

47. Mangham LJ, Hanson K, McPake B. How to do (or not to do) …Designing a discrete choice experiment for application in a low-income country. Heal Policy Plan [Internet]. 2009;24. Available from: https://doi.org/10.1093/heapol/czn047

48. Sawtooth Software. CBC vs.ACBC: Comparing Results with Real Product Selection. 2007. p. 8.

49. Waylon B, Samuel S, Mitchell S, Clarice L, Jeffrey B, Anderson Richard. Open Data Kit 2.0: A Services-Based Application Framework for Disconnected Data Management [Internet]. 2017 [cited 2019 Aug 8]. Available from: http://delivery.acm.org/10.1145/3090000/3081365/p440-brunette.pdf?ip=129.56.77.214&id=3081365&acc=OA&key=4D4702B0C3E38B35.4D4702B0C3E38B35.4D4702B0C3E38B35.BBCD68C0FAC0CC7C&__acm__=1565441939_69a8e17ae191b89ed0d00dc9d678c033

50. de Bekker-Grob EW, Donkers B, Jonker MF, Stolk EA. Sample Size Requirements for Discrete-Choice Experiments in Healthcare: a Practical Guide. Patient - Patient-Centered Outcomes Res [Internet]. 2015 Oct;8(5):373–84. Available from: https://doi.org/10.1007/s40271-015-0118-z

51. Lancsar E, Louviere J. Conducting discrete choice experiments to inform healthcare decision making: a user's guide. Pharm Econ [Internet]. 2008;26. Available from: https://doi.org/10.2165/00019053-200826080-00004

52. Orme B. Sample Size Issues for Conjoint Analysis. Sequim Sawtooth Softw Tech Pap. 1998;

53. Johnson R, Orme B. Getting the most from CBC. Sequim Sawtooth Softw Res Pap Ser Sawtooth Softw. 2003;

54. STATACorp. State statistical software. College Station, TX: STATACorp LLC; 2019.

For peer review only

Authors' contributions

SA conceptualized the study and led the writing of the first draft of the protocol. UA assisted with relevant background search and writing. CEW designed the overall frontline health worker study, TA, RK, AC, SHadapted the protocol for Kenya, Uganda, Haiti and Bangladesh, respectively. All authors supported the development of the research design and reviewed the final manuscript.

Funding statement

This study is funded by the Bill & Melinda Gates Foundation. The funding body has no role in the study design, data collection, analysis, or interpretation of data.

Conflict of interest's statement

The authors declare no conflict of interests

Data statement

Data can be made available on request after the initial analysis is completed and disseminated.

For peer review only

BMJ Open

Understanding Incentive Preferences of Community Health Workers using Discrete Choice Experiments: A multi-country protocol for Kenya, Uganda, Bangladesh and Haiti

Journal:	*BMJ Open*
Manuscript ID	bmjopen-2019-033601.R1
Article Type:	Protocol
Date Submitted by the Author:	16-Oct-2019
Complete List of Authors:	Agarwal, Smisha ; Johns Hopkins University Bloomberg School of Public Health<br>Anaba, Udochisom; Population Council<br>Abuya, Timothy; Population Council, Reproductive Health Program<br>Kintu, Richard; Pathfinder International<br>Casseus, Alain; Zanmi Lasante<br>Hossain, Sharif; Population Council<br>Obadha, Melvin; KEMRI<br>Warren, Charlotte; Population Council
**Primary Subject Heading**:	Global health
Secondary Subject Heading:	Health policy, Global health, Health services research
Keywords:	Community Health Workers, Community Health Systems, Incentives, Discrete Choice Experiment

SCHOLARONE™
Manuscripts

Understanding Incentive Preferences of Community Health Workers using Discrete Choice Experiments: A multi-country protocol for Kenya, Uganda, Bangladesh and Haiti

Authors:

Smisha Agarwal (smagarwa@jhsph.edu), John Hopkins Bloomberg School of Public Health, 615 N Wolfe Street, Baltimore 21205, Maryland, United States (Corresponding author)

Udochisom Anaba (uanaba@popcouncil.org), Population Council, 4301 Connecticut Ave NW # 280, Washington, DC 20008, United States

Timothy Abuya (tabuya@popcouncil.org), Population Council, Avenue 5, 3rd floor, Rose Avenue, Nairobi, Kenya

Richard Kintu (rkintu@pathfinder.org), Pathfinder International, Plot 30B, Impala Ave, Kololo, Kampala, Uganda

Alain Casseus (acasseus@pih.org), Zanmi Lasante, Santo 18A, Croix-des-Bouquets, Haiti

Sharif Hossain (sharifhossain@popcouncil.org), Population Council, House #12, Road #25/30 Gulshan-1 , Dhaka 1212, Bangladesh

Melvin Obadha (MObadha@kemri-wellcome.org), Health Economics Research Unit, KEMRI-Wellcome Trust Research Programme, Nairobi Kenya

Charlotte E Warren (cwarren@popcouncil.org), Population Council, 4301 Connecticut Ave NW # 280, Washington, DC 20008, United States

Corresponding Author:

Smisha Agarwal
Email: smagarwa@jhsph.edu
Address: Suite W5009E, 615 N Wolfe Street, Baltimore, Maryland, USA
Tel No: +1-347-768-2070

Word count: 4067

For peer review only

For peer review only

Abstract

Introduction

There is a renewed global interest in improving community health worker (CHW) programs. For CHW programs to be effective, key intervention design factors which contribute to the performance of CHWs need to be identified. The recent World Health Organization guidelines recommends the combination of financial and non-financial incentives to improve CHW performance. However, evidence gaps remain as to what package of incentives will improve their performance in different country contexts. This study aims to evaluate CHW incentive preferences to improve performance and retention which will strengthen CHW programs and help governments leverage limited resources appropriately.

Methods and analysis

A discrete choice experiment (DCE) will be conducted with CHWs in Bangladesh, Haiti, Kenya and Uganda with different levels of maturity of CHWs programs. This will be carried out in two phases. Phase I will involve preliminary qualitative research including focus group discussions and key informant interviews to develop the DCE design which will include attributes relevant to the CHW country settings. Phase II will involve a DCE survey with CHWs, presenting them with a series of job choices with varying attribute-levels. An orthogonal design will be used to generate the choice sets for the surveys. The surveys will be administered in locally-appropriate languages to at least 150 CHWs from each of the cadres in each country. Conditional and Mixed Multinomial logit models will be used for the estimation of stated preferences.

Ethics and dissemination

This study has been reviewed and approved by the Population Council's Institutional Review Board in New York, and appropriate ethics review boards in Kenya, Uganda, Bangladesh and Haiti. The results of the study will be disseminated through, in-country dissemination workshops, meetings with country-level stakeholders and policy working groups, print media, online blogs, and peer-reviewed journals.

For peer review only

Keywords

Community Health Workers, Community Health Systems, Incentives, Discrete Choice Experiment, Performance

For peer review only

Strengths and limitations of this study

- Assessment of multi-stakeholder perspectives to identify important job attributes will provide policy relevant and actionable choice options to be tested in the study
- Draws evidence from multiple countries with strong country-level stakeholder involvement including ministries of health, non-governmental organizations and donor agencies
- In countries with multiple cadres of CHWs, the study includes formative interviews with at least two cadres of CHWs to understand their perspectives
- As with other discrete choice experiments, a key limitation is that the job alternatives presented to the CHWs are hypothetical.
- The results have limited generalizability across the countries given the variation that tends to exist in the needs of CHWs across cadres, terrain and job responsibilities.

For peer review only

Introduction

The dearth of health workers, especially in areas where they are most needed, continues to be an ongoing challenge for health systems globally. The World Health Organization (WHO) forecasted a global shortage of approximately 18 million health workers by 2030.(1) Recruitment and training of community health workers (CHWs) provides tremendous potential to address this gap in human resources as well as ensure access to basic health services where the formal sector falls short.(2,3) The Alma Ata Declaration (1978), considered a critical twentieth century public health milestone, highlighted the key role of CHWs in advancing "health for all".(4) Consequently, CHW programs became the cornerstone for rapidly expanding primary healthcare services for poor populations.(5) Over the 1980's and 1990's, several pilot CHW programs reported substantial improvements in healthcare outcomes, demonstrating the potential contribution of CHWs.(6,7) However, most national level CHW programs were variably implemented and reported several challenges that limited the effectiveness of these programs.(8–10) Scaled CHW programs had high levels of attrition and overall reduced quality of care. It was argued that the poor performance of these national level programs was not due to the failure of the concept of CHWs, but the lack of ongoing training, supervision, logistical and financial support, as well as weaker linkages to the health system.(11) Often CHWs were unpaid volunteers and were not accountable to the health system. Where strategies to expand care through CHWs was employed as an alternative rather than a complement to professional care, the results were poor.(12) Several debates about selective versus comprehensive PHC approaches ensued.(10) The recent Astana Declaration (2018) has once again emphasized the critical role of primary health care in advancing Universal Health Coverage (UHC).(13) Once again, the potential contribution of CHWs to supporting UHC has come to the forefront.

As governments respond to this wave of renewed enthusiasm for CHW programs, it is critical to be responsive to the lessons from the past. Evidence from studies conducted over the past five decades have identified significant contributions as well as challenges of CHW programs. Bhutta et al. conducted a

For peer review only

systematic review of evidence to recommend several behavioral and promotional interventions which can be effectively delivered by CHWs with limited training or trained CHWs along the continuum of maternal health care. These include the promotion of reproductive health services and family planning, appropriate care seeking and antenatal care during pregnancy, and skilled care for childbirth.(14,15) Additionally, CHWs can play a critical role in several preventive and treatment interventions to support adequate maternal and child nutrition and vitamin supplementation, identification of high-risk pregnancies and childhood illnesses, and early management of preterm labor, malaria in pregnancy, infections and malnutrition in children.(14) However, it is also well-established that CHW programs can only be effective when CHWs are appropriately recruited, sufficiently trained and adequately supported. Understanding the preferences of CHWs is core to facilitating their retention and improved performance. Broadly, factors affecting the motivation and performance of CHWs can be categorized as: Intervention design factors that include but are not limited to incentives, training, supervision, monitoring & evaluation mechanisms; (16–18) and contextual factors like the community context, socio-cultural factors, work environment, economic context and health system policy and practice.(15,19,20) While there is some research on the programmatic inputs that contribute to strong CHW programs, further research on the appropriate combination of these inputs can help governments leverage limited resources appropriately. The 2018 WHO guideline on health policy and system support to optimize CHW programs identified this as a key research gaps and concluded that "evidence is not sufficiently granular to allow recommendation of specific forms of interventions, for example….which bundle of financial and non-financial incentives are most effective".(21)

In many LMICs, CHWs are often funded by government or donors.(22,23) Historically, the commitment to consistently fund comprehensive CHWs programs has wavered; often, in preference for vertical programs.(19) While in the early years CHWs were thought of as "volunteers", the importance of integrating these health works into the formal health sector, including the provision of adequate remuneration as well as other incentives has been widely researched and documented.(24–26) The 2018 WHO guideline on CHWs recommended that practicing CHWs are remunerated for their work "with a

For peer review only

financial package commensurate with the job demands, complexity, number of hours, training and roles that they undertake". It further elaborated that incentives can include financial remuneration, including performance-based incentives, as well as non-financial incentives.(21) This was also captured in the Astana Declaration as "We will create decent work and appropriate compensation for health professionals and other health personnel working at the primary health care level………… continue to invest in the education, training, recruitment, development, motivation and retention of the PHC workforce".(13)

However, barriers to instituting these recommendations in practice remain. First, there is no agreement on strategies that would best support adequate incentivization of CHWs. Second, with respect to health worker compensation, the health sector human resource policies and practices vary considerably across countries. Third, given the optimal ratio of CHW to population, the question of affordability of scaled CHW programs to governments remains. A study of CHW program costs in 37 sub-Saharan African countries estimated that when CHWs are paid an equivalent of US$80/month by existing central government healthcare budgets, the median relative cost of a CHW program would be 27 percent of the national healthcare budget. (27)

This study aims to address this critical gap in understanding the combination of financial and non-financial incentives that will support CHWs, improve their work satisfaction and retention, and consequently influence the performance of CHW programs. The proposed study is being conducted by Frontline Health Project through Population Council, and Johns Hopkins Bloomberg School of Public Health, supported by Bill and Melinda Gates Foundation, in partnership with USAID, UNICEF and Integrating Community Health (ICH) NGO partners in Bangladesh, Uganda, Kenya and Haiti. The Frontline Health project works with global stakeholders to contribute to the objective of advancing evidence for community health. Given the active leadership and participation of donors, NGOs and governments for this study, we expect that the results will aid donors and national policymakers to develop training and remuneration policies that are conducive to high performance of CHW programs, as well as maximize the effective use of limited resources.

For peer review only

The theoretical underpinning of our approach comes from evidence that suggests that the context within which CHW work-including socio-cultural factors, support from the health system, relationship with the community, respect and recognition, among others- all contribute to their motivation to work as CHWs, and subsequently their performance. (19,28) For the purpose of this study, we consider these factors, as well as any direct financial compensation as "incentives". To elicit the incentive preferences of CHWs, we propose the use of discrete choice experiments (DCEs). DCEs present respondents with two or more hypothetical choices and ask them to select their preferred alternatives.(29) The alternatives are defined by two or more attributes and levels. In this study, we propose to present CHWs with alternatives with different job attributes (e.g. salary, promotion, recognition etc.) that have been systematically identified through stakeholder interviews and a review of literature. Based on the selection of preferred alternatives, the influence of each attribute on the choice can be estimated.(30) Furthermore, trade-offs respondents are willing to make can be quantified specifically the marginal willingness to accept (WTA) monetary compensation.

DCEs are based on Lancaster's consumer demand theory and Random Utility Theory (RUT). According to Lancaster's theory, individuals derive benefit or value (utility) from the attributes of a good or service under consideration rather than the good/service itself.(31) (32) RUT generally suggests that individuals are rational decision makers aiming to solve an optimization problem. They assign perceived utility to each alternative and will therefore choose the one that derives them the highest utility among a set of mutually exclusive alternatives.(33–35) These utilities are contingent on the attributes of the alternative and the characteristics of the decision maker. However, utilities are latent but researchers can draw conclusions about them from the choices respondents make in a DCE survey.

This approach has been used in comparable studies involving CHWs in India and healthcare workers in LMICs.(36,37) Prior studies have used DCE to model different policy interventions on the recruitment of

For peer review only

nurses to rural areas in Kenya, South Africa and Thailand (38). In Mozambique, DCE was used to elicit the job preferences of non-physician health professionals (39). In Tanzania and rural Vietnam, DCE has been used to understand how to make jobs in rural areas more attractive to medical students and doctors respectively.(40,41) In Uganda, DCE was used with volunteer CHWs in family planning programs in 2011 to examine factors related to their motivation. The study identified recognition in form of t-shirts and badges, a mobile phone and social prestige as some of the core elements associated with CHW motivation.(42,43)

Methods and Analysis

We will use a DCE to examine the job attributes that influence the overall satisfaction and motivation of CHWs in Bangladesh, Haiti, Kenya and Uganda. For the study, we focus on a set of factors that are amenable to national policy and programmatic changes. The study will be conducted in two phases. In the first phase, we aim to identify a set of attributes that are realistic, pragmatic and meaningful to CHWs and policymakers, and in the second phase, we will prioritize critical attributes associated with job satisfaction from the CHW's point of view. The study design considers the varying context across the four countries, as well as the multiple cadres and roles of the CHWs in each country. The study will be conducted between June 2019-October 2020.

Study setting and participants

Bangladesh has 8 administrative divisions, 64 districts, and 491 sub-districts (Upazilas). Each rural area within an upazila is divided into union parishads and mouzas, which are further divided into villages. An urban area in an upazila is divided into wards, and mohallas within each ward.(44) The smallest units within rural areas are the villages and CHWs operate at this level. Bangladesh has several cadres of health workers operating at the community-level. Health Assistants (HA), Family Welfare Assistants (FWA) and Community Health Care Providers (CHCP) are commonly referred to as CHWs in the public sector, and they work within the purview of primary health care centers known as Union Health and Family Welfare

For peer review only

Centers. Currently, the government is also recruiting multipurpose volunteers and prepaid volunteers in areas where vacancies and workload is higher. In addition, there are CHWs in the non-governmental systems with various names. For example, BRACs CHWS include the *Shasthya Shebikas*, which has been one of the largest cadres of CHWs in Bangladesh. However, currently the scale and function of the *Shasthya Shebikas* is being revised owing to the high attrition rate of these workers. (45)

Based on discussions with the Directorate General of Family Planning (DGFP) and Directorate General of Health Services (DGHS), the focus of the study will be on the two government-supported CHW cadres- FWAs (supported by the DGFP) and HAs (supported by the DGHS). The study will be conducted in the four Divisions of Sylhet, Rajshahi and Khulna, as well as in Chittagong Division's Cox's Bazaar area. The Upazilas within each Division will be identified through further discussions with the DGFP and DGHS.

Haiti is divided into 10 geo-political regions and sub-divided into 42 states (arrondisements).(46) Each arrondisement is further divided into 142 communes in total which comprises 571 communal sections. The health system is overseen at the national level by the Ministry of Public Health and Population (MSPP) which provides policies and standards for service delivery at all levels (including at community level). The District Health Unit, supported by the Health Department Directorate, coordinates, supervises and monitors care delivery at community level.(47) The primary community health providers are the "agents de santé communautaire polyvalent" or ASCP which have been working informally in Haiti for years, but were more formally established by the MSPP in 2015 under one title and clear job description.(47)

This study will take place in three communes located in two arrondisements - one in Artibonite region and the other in Centre region. In Centre region, the study will occur in Mirebalais commune, Mirebalais arrondisement. In Artibonite region, the study will occur in Petite Riviere de L'Artibonite (PRA) commune and Verettes commune. Study sites and communal sections were selected in collaboration with Zanmi Lasante who have a strong collaboration and presence with the ASCPs in Haiti as well as the MSPP.

For peer review only

Kenya is made up of 47 counties with a population projection of more than 40 million. The healthcare system in Kenya is based on the 2010 Kenyan Constitution (48) which operates a decentralized system of government with the National government and 47 county governments. Kenya has a hierarchical four-tier healthcare system at the community, primary care, secondary referral and tertiary referral levels with Community Health Volunteers (CHVs) operating at the community level. The community services comprise all community-based demand creation activities and health services organized around a comprehensive community strategy defined for the health sector. The primary care services comprise all dispensaries, health centres, and maternity and nursing homes in both public and private sectors. The county referral services include hospitals operating in and managed by a county both public and private which form the county referral system.(49) Every 1,000 household (approx. 5,000 people) are covered by one Community Health Unit (CHU), comprising of a Community Health Committee (CHC) and 5 of Community Health Extension Workers (CHEWs). CHEWs supervise Community Health Volunteers (CHVs) who are embedded within community health units (CHUs). CHVs are supposed to be supervised at the community level by community health committees (CHCs).

In Kenya, the study will take place in Bungoma and Kilifi counties. For phase one, CHVs, CHEWs county level community focal person and the national stakeholders who works closely with CHV will be interviewed. For phase two CHVs will be the primary survey respondent in the two counties. Kilifi is a coastal county with mixed rural urban populations, and Bungoma is an agrarian county in Western Kenya. These counties represent a set of diverse vulnerabilities within which CHWs operate. Within each of these counties, two sub-counties will be selected in consultation with the county health management teams.

In Uganda, the Health Sector Development Plan highlights the critical shortage of health workers, with a ratio of 1.55 health workers per 1000, compared to the WHO recommendations of 2.28 per 1000. Since 2001, in Uganda, Village Health Teams (VHT) were established to support community health through

health promotion and education activities, mobilization of communities for utilization of health services, and treatment of illnesses at the community-level. In 2014, based on a comprehensive VHT assessment, it was recommended that the VHT strategy is overhauled, along with consideration of a standardized approach for incentives for all VHTs.(50) In response to these findings, the Ministry of Health is in the process of discussing a new Community Health Extension Worker (CHEW) strategy in the recent years. Under this new strategy, CHEWs will be adequately trained in promotive and basic curative health services, and their role will encompass supervising the VHTs. However, at the time of conducting the study, the CHEW policy is pending cabinet approval. In addition to the VHTs, Uganda also have CHWs that are supported by non-governmental organizations. Hence, for this study, we focus on two cadres in Uganda- CHWs and VHTs. CHWVHTs, policymakers and CHW/VHT supervisors will be interviewed during phase 1. Based on detailed discussions with the MoH and other stakeholders, the study will be conducted across eight districts — Lira, Mayuge, Wakiso, Ntungamo, Kabale, Arua, Kabarole and Nakapiripiriti — to facilitate greater representation of the cadres of CHWs/VHTs across the country. These counties were selected based on considerations for the types of terrain and tribes where CHWs serve.

Phase 1: Sampling and approach to inform the development of attributes and levels

In the first phase, an initial set of policy-relevant, realistic and actionable attributes will be identified based on a review of secondary literature, as well as primary research involving focus group discussions (FGDs) with a sample of CHWs, their supervisors, and national-level stakeholders involved in making decisions about CHW programs in the country. The attributes and attribute-levels will be established separately for each country, based on information collected through the literature review and this formative phase. Discussion topics will include, but not be limited to, expected remuneration, working conditions, availability of supplies and equipment, amount of training, supervision, job role and expected support from the health system and community. Participants will be probed to identify additional priority areas. The FGD will use a nominal group technique where the facilitator will ask the participants to think about aspects of the job that are most important to CHWs as well as actionable by policymakers. Participants will silently

For peer review only

generate a list of attributes, and then be asked to state a single attribute to the group.(51) This will be recorded on a board. This will continue until saturation of attributes is reached. Following this, a discussion to identify the levels of the attributes will be initiated. Participants will be asked to rank order the identified attributes and their levels privately based on their preferences. While there are no restrictions on the number of attributes that can be included in a DCE, in practice, fewer than 10 attributes are selected to reduce cognitive burden on the respondents thereby avoiding inconsistent responses, lexicographic behavior, and attribute non-attendance (where respondents do not consider all attributes in making their decisions).(52) Definitions of each attribute will be defined, appropriate to the setting, and any overlap or correlation between the attributes will be avoided. Base levels for each attribute will be established to reflect the prevailing working conditions in the country for government-supported CHWs. Additional levels will be then determined to represent a reasonable improvement from the base level. Levels will be chosen to reflect a range of situations that CHWs might realistically expect to experience.

An estimated number of 4 FGDs with 5-7 national and county/subcounty level policy makers, 4-6 FGDs with CHW supervisors and ~6-8 FGDs with CHWs (each cadre) will be conducted in each country. These numbers will vary with the context in each country with the categories of actors involved with community health system as well as the sub regional levels where the study will be conducted. Additionally, for policymakers who cannot be convened in a group setting, the same set of interview questions will be asked to them as key informants in an in-depth interview format. All participants must be aged 18 years or and with the ability to provide consent. There are no eligibility criteria related to gender or marital status. Policymakers will be identified by the study team and other relevant partners and CHWs and their supervisors will be recruited through community health units/primary health care facilities in select counties/districts. To ensure that the study takes a ground-up approach, the first set of interviews will be conducted with CHWs and supervisors. The study team will review the results of these interviews to then align the discussion with the policymakers and other stakeholders to reflect the priorities of the CHWs.

For peer review only

Prioritizing attributes and determining choice sets

Attributes and levels identified in phase 1 across all the interviews will be finalized by the study team based on the discussion transcripts. To ensure that the final set of attributes and levels represent a pragmatic and desirable set, they will be further vetted with external stakeholders such as ministry representatives and non-governmental organization leaders working in the CHW space. Efforts will be made to have clear definitions of each attributes and avoid conceptual overlaps across attributes and levels.

A fractional factorial design, which only takes a subset of choice sets, will be generated as too many choice sets place cognitive burden on respondents.(29) An orthogonal design will be generated using Sawtooth software Inc (Sequim, WA, USA).(53) where each attribute will be independent of each other and each attribute- level will occur equally often (balanced.(35)

Sawtooth software Inc. To generate the experiment will be unlabeled, full profiles, and consist of three alternatives i.e. Two job alternatives and an opt-out option.

The DCE questionnaire will include questions about respondent demographics, content and years of training as a CHW, socio-economic status, and current level and quality of supervision. The order of the choice sets will be randomized to avoid positional bias. In each questionnaire, a dominant alternative will be included where one job option is superior to the other on all characteristics so that internal consistency and rationality of the responses can be considered during analysis.(29) Finally, the questionnaires will be pre-tested with a sub-set of 20-30 respondents and changes made to the content and wording of the questions to account for any conceptual overlap and lack of clarity.

Phase 2: Sampling and approach for the DCE

In each country, the questionnaire will be translated into local language/s. The survey will be administered face-to-face to the CHWs by trained data collectors on tablets using the Open Data Kit (ODK) software (54). First, the purpose of the study will be explained. The data collectors will explain the meaning of each attribute to the CHWs to support comprehension of the choice tasks. Each CHW will consider a set of job

For peer review only

alternative and will be asked to choose an option they prefer . They will also have to option to opt-out i.e. choose neither of the job alternative presented to them.

Sample size calculation in DCE studies mostly rely on rule-of-thumb estimates.(51) For estimation of a DCE model, Lancsar & Louviere (30) suggested that if respondents receive the same design, one rarely requires more than a sample size of 20 respondents. Other studies have shown that sample sizes of 40–100 respondents or up to 300 respondents may be adequate for reliable statistical analysis.(55) The sample size calculation for this study is guided by the rule-of-thumb approach proposed by Johnson and Orme, as well as expected number of choice sets and pragmatic considerations around time and budget to conduct the study across the four countries.(56,57). This approach was selected as it provides a better method of sample size calculation over other techniques such as those proposed by Lancsar and Louviere. According to Johnson and Orme, the minimum sample size *N* needed to detect differences in the preferences is dependent on the number of analysis cells *c,* number of choice sets *t,* and number of alternatives *a* as shown in the following equation;

$$N > 500c \,/\, (t \times a)$$

Assuming a main effects model, then *c* will be equal to the largest number of levels for any of the attributes. If we assume that respondents will face a maximum of 18 choice sets, the largest number of levels of any attribute in a main effects model is four, and that each task has two alternatives (excluding the opt-out), then using equation 1, we derive a minimum sample size of 56. Therefore, we estimate that recruiting a minimum of 150 CHW per cadre per country to be more than sufficient to detect differences in preferences. A total of approximately 900 respondents across the four countries will be recruited for Phase 2 (Bangladesh-300; Haiti-150; Kenya- 150; Uganda-300). Based on the total sample size, an equal number of CHWs will be recruited per geographic area in discussion with the county/district health management teams to get broad representation of the types of CHWs.

Public and Patient involvement

For peer review only

The first phase of the study is structured to formally gather feedback from the broader community that supports CHWs, including national and international stakeholders and donors, other health officials, CHW supervisors and CHWs themselves. Opinions and experiences of these stakeholders are critical to shaping the questionnaire design and approach for the second phase of the study. Health officials at the facilities will be involved in the identification and recruitment of the CHWs in Phase 2 of the study. Study results will be disseminated through in-country workshops where health officials involved during the design and recruitment phase will be invited to participate.

Analysis Plan

Conditional logit model will be used as the base model as it is congruous with RUT .(33) This will model the probability of choosing one job alternative over another given the differences in the attribute levels. Preference heterogeneity will be explored by introducing interactions between the attributes. Furthermore, the relative importance CHWs place on the job attributes will be quantified. Additionally, where feasible, marginal WTA estimates will be computed which is the amount of money CHWs are willing to accept to compensate for the worsening or improvement of an attribute.(35) The monetary attribute will be monthly salary while for volunteer CHWs, it will be stipends.

However, one of the short comings of the conditional logit model is that the assumption of the independence of irrelevant alternatives (IIA) must hold. This is as a result of the error terms being assumed to be independently and identically distributed (IID) following extreme value type 1 (Gumbel) distribution. To relax IIA and further explore preference heterogeneity, a Mixed Multinomial Logit model (MMNL) will be used. MMNL presumes that some or all the parameters are randomly distributed with a particular probability distribution.(33) MMNL will result in means which represent the choice probabilities and standard deviations which denote heterogeneity. Stata 16 software will be used in the analysis (58)

Limitations of the study in this context

For peer review only

Intrinsic and extrinsic factors influencing CHW incentive preferences are diverse and real-life decisions change over time, therefore the relative importance of job preferences may vary with time and this needs to be considered when designing CHW programs and policy interventions. We also recognize that CHW preferences can be different based on their organizational affiliations, and prior experiences working as CHWs. While the study aims to capture a sample of CHWs across few areas in each country, the sample is not nationally representative of the preferences of all CHWs. We recognize that a comprehensive job package for CHWs may comprise of many more factors than can be adequate captured by this study design. Finally, the results of this study will constitute stated preferences, reflecting responses to hypothetical scenarios. Decision makers will need to consider information from DCEs on CHW as part of the inputs to improved CHW performance and retention while making policies.

For peer review only

Ethics and dissemination

This study has been reviewed and approved by the Population Council's Institutional Review Board in New York, the AMREF-Health Africa Ethics and Scientific Review Committee in Kenya, Zanmi Lasante's IRB (Haiti) and the Higher Degrees Research Ethics Committee at Makerere University College of Health Sciences in Uganda, and the Bangladesh Medical Research Council (BMRC). Dissemination activities will include sharing of findings through, policy briefs, workshops, online blogs in community fora, and peer-reviewed journal articles. The research approach is inherently designed around the need for research uptake by policy-makers. Involvement in the research from the beginning is the over-arching strategy for promoting research uptake. To ensure there is significant demand for the final study findings, we will debrief policy makers and other health system stakeholders at pivotal points in the initial stages as well as at the end of the study. We will share non-technical summaries of research results with all the partners for dissemination including the study's key partners in the Ministry of Health. If there is enough interest, appropriate dissemination will be conducted with policymakers in half-day workshops, and to facilitate translation of the research findings in national CHW agenda.

For peer review only

References

1. Darzi A, Evans T. The global shortage of health workers-an opportunity to transform care. Lancet (London, England). 2016 Nov;388(10060):2576–7.

2. Perry HB, Zulliger R, Rogers MM. Community Health Workers in Low-, Middle-, and High-Income Countries: An Overview of Their History, Recent Evolution, and Current Effectiveness. Annu Rev Public Heal. 2014;35:399–421.

3. O'Donovan J, O'Donovan C, Kuhn I, Sachs SE, Winters N. Ongoing training of community health workers in low-income and middle-income countries: a systematic scoping review of the literature. BMJ Open. 2018 Apr;8(4).

4. World Health Organisation. Declaration of Alma-Ata: International Conference on Primary Health Care, Alma-Ata, USSR, 6-12. 1978.

5. Christopher JB, Le May A, Lewin S, Ross D a. Thirty years after Alma-Ata: a systematic review of the impact of community health workers delivering curative interventions against malaria, pneumonia and diarrhoea on child mortality and morbidity in sub-Saharan Africa. Hum Resour Health [Internet]. 2011;9(1):27. Available from: http://www.human-resources-health.com/content/9/1/27

6. Bang AT, Bang RA, Baitule SB, Reddy MH, Deshmukh MD. Effect of home-based neonatal care and management of sepsis on neonatal mortality: field trial in rural India. Lancet (London, England) [Internet]. 1999 Dec 4 [cited 2016 Apr 6];354(9194):1955–61. Available from: http://www.sciencedirect.com/science/article/pii/S0140673699030469

7. Chowdhury AM, Chowdhury S, Islam MN, Islam A, Vaughan JP. Control of tuberculosis by community health workers in Bangladesh. Lancet (London, England) [Internet]. 1997 Jul 19 [cited 2016 Apr 6];350(9072):169–72. Available from: http://www.sciencedirect.com/science/article/pii/S0140673696113118

8. Gilson L, Walt G, Heggenhougen K, Owuor-Omondi L, Perera M, Ross D, et al. National community health worker programs: How can they be strengthened? J Public Health Policy. 1989;10(4):518–32.

9. Frankel S. The community health worker: effective programmes for developing countries. Oxford University Press; 1992.

10. Rosato M, Laverack G, Grabman LH, Tripathy P, Nair N, Mwansambo C. Community participation: lessons for maternal, newborn, and child health. Lancet. 2008;372.

11. Lawn JE, Lawn JE, Rohde J, Rohde J, Rifk S, Rifk S, et al. Series Alma-Ata: Rebirth and Revision 1 Alma-Ata 30 years on: revolutionary, relevant, and time to revitalise. Lancet, The. 1978;917–27.

12. World Health Organization. Primary health Care - Now more than ever. [Internet]. Vol. 26, The World Health Report. 2008. Available from: http://www.who.int/whr/2008/whr08_en.pdf

13. World Health Organisation & UNICEF. Declaration of Astana: Global Conference on Primary Healtcare. 2018.

14. Bhutta ZA, Ali S, Cousens S, Ali TM, Haider BA, Rizvi A, et al. Interventions to address maternal, newborn, and child survival: what difference can integrated primary health care

For peer review only

strategies make? Lancet. 2008;372(9642):972–89.

15. Haines A, Sanders D, Lehmann U, Rowe AK, Lawn JE, Jan S. Achieving child survival goals: potential contribution of community health workers. Lancet. 2007;369.

16. Nsabagasani X, Jesca Nsungwa S, Kallander K, Peterson S, Pariyo G, Tomson G. Home-based management of fever in rural Uganda: community perceptions and provider opinions. Malar J. 2007;6.

17. Puett C, Coates J, Alderman H, Sadler K. Quality of care for severe acute malnutrition delivered by community health workers in southern Bangladesh. Matern Child Nutr. 2013;9.

18. Takasugi T, Lee ACK. Why do community health care workers volunteer: a qualitative study in Kenya. Public Health. 2012;126.

19. Kok MC, Kane SS, Tulloch O, Ormel H, Theobald S, Dieleman M, et al. How does context influence performance of community health workers in low- and middle-income countries? Evidence from the literature. Heal Res Policy Syst. 2015 Mar;13(1):13.

20. Viswanathan K, Hansen PM, Hafizur Rahman M, Steinhardt L, Edward A, Arwal SH. Can community health workers increase coverage of reproductive health services? J Epidemiol Community Heal. 2012;66.

21. WHO. Guideline on health policy and system support to optimize community health worker programmes. 2018. p. 1–116.

22. Kok MC, Ormel H, Broerse JEW, Kane S, Namakhoma I, Otiso L, et al. Optimising the benefits of community health workers' unique position between communities and the health sector: A comparative analysis of factors shaping relationships in four countries. Glob Public Health. 2017;12(11):1404–32.

23. Phiri SC, Prust ML, Chibawe CP, Misapa R, van den Broek JW, Wilmink N. An exploration of facilitators and challenges in the scale-up of a national, public sector community health worker cadre in Zambia: A qualitative study. Hum Resour Health. 2017 Jun;15(1):40.

24. Karabi B, Leban K, Winch P, Tien M. Community Health Worker Incentives and Disincentives: How They Affect Motivation, Retention, and Sustainability. Basic Support for Institutionalizing Child Survival Project (BASICS II) for the United States Agency for International Development; 2001.

25. Singh D, Negin J, Otim M, Orach CG, Cumming R. The effect of payment and incentives on motivation and focus of community health workers: five case studies from low- and middle-income countries. Hum Resour Health. 2015;13(1):58.

26. Scott K, Beckham SW, Gross M, Pariyo G, Rao KD, Cometto G, et al. What do we know about community-based health worker programs? A systematic review of existing reviews on community health workers. Hum Resour Health. 2018 Aug;16(1):39.

27. Taylor C, Griffiths F, Lilford R. Affordability of comprehensive community health worker programmes in rural sub-Saharan Africa. BMJ Glob Heal. 2017 Sep;2(3):e000391.

28. Mpembeni RNM, Bhatnagar A, LeFevre A, Chitama D, Urassa DP, Kilewo C, et al. Motivation and satisfaction among community health workers in Morogoro Region, Tanzania: nuanced needs and varied ambitions. Hum Resour Health. 2015;13:44.

29. Mangham LJ, Hanson K, McPake B. How to do (or not to do) …Designing a discrete choice

For peer review only

experiment for application in a low-income country. Heal Policy Plan. 2009;24.

30. Lancsar E, Louviere J. Conducting discrete choice experiments to inform healthcare decision making: a user's guide. Pharm Econ. 2008;26.

31. Lancaster KJ. A New Approach to Consumer Theory. J Polit Econ. 1966 Apr;74(2):132–57.

32. Robyn PJ, Shroff Z, Zang OR, Kingue S, Djienouassi S, Kouontchou C, et al. Addressing health workforce distribution concerns: a discrete choice experiment to develop rural retention strategies in Cameroon. Int J Heal Policy Manag. 2015;4(3):169–80.

33. Daniel McFadden. Conditional logit analysis of qualitative choice behavior. In: Frontiers in Econometrics. P.(ed). New York, NY: Academic Press; 1973.

34. Amaya-Amaya M, Gerard K, Ryan M. Discrete choice experiments in a nutshell. Ryan M, Gerard K, Amaya-Amaya M, editors. Dordrecht: Springer; 2008.

35. Hensher DA, Rose JM, Greene WH. Applied Choice Analysis. 2nd ed. Cambridge: Cambridge University Press; 2015.

36. Shiratori S, Agyekum EO, Shibanuma A, Oduro A, Okawa S, Enuameh Y, et al. Motivation and incentive preferences of community health officers in Ghana: an economic behavioral experiment approach. Hum Resour Health. 2016;14(1):53.

37. Abdel-All M, Angell B, Jan S, Howell M, Howard K, Abimbola S, et al. What do community health workers want? Findings of a discrete choice experiment among Accredited Social Health Activists (ASHAs) in India. BMJ Glob Heal. 2019;4(3).

38. Blaauw D, Erasmus E, Pagaiya N, Tangcharoensathein V, Mullei K, Mudhune S, et al. Policy interventions that attract nurses to rural areas: a multicountry discrete choice experiment. Bull World Health Organ. 2010;88:350–6.

39. Honda A, Vio F. Incentives for non-physician health professionals to work in the rural and remote areas of Mozambique---a discrete choice experiment for eliciting job preferences. Hum Resour Health. 2015 Apr;13(1):23.

40. Kolstad J. How to make rural jobs more attractive to health workers. Findings from a discrete choice experiment in Tanzania. Health Econ. 2010;20.

41. Vujicic M, Alfano M, Shengeliac B, Witter S. Attracting Doctors and Medical Students to Rural Vietnam: Insights from a Discrete Choice Experiment. Washington, D.C: World Bank; 2010.

42. Brunie A, Chen M, Practice AA-GHS and, 2016 undefined. Qualitative Assessment of the Application of a Discrete Choice Experiment With Community Health Workers in Uganda: Aligning Incentives With Preferences. ghspjournal.org [Internet]. [cited 2019 Oct 14]; Available from: http://www.ghspjournal.org/content/4/4/684?utm_source=TrendMD&utm_medium=cpc&utm_campaign=Global_Health%253A_Science_and_Practice_TrendMD_1

43. Brunie A, Wamala-Mucheri P, Otterness C, Akol A, Chen M, Bufumbo L, et al. Keeping community health workers in Uganda motivated: key challenges, facilitators, and preferred program inputs [Internet]. [cited 2019 Oct 14]. Available from: www.ghspjournal.org

44. Ahmed S, Alam B, Anwar I, Begum T, Huque R, Khan J, et al. Bangladesh Health System Review. Manila; 2015.

45. Ahmed SM, Evans TG, Standing H, Mahmud S. Harnessing pluralism for better health in

For peer review only

Bangladesh. Lancet. 2013;382(9906):1746–55.

46. World Bank. Country Profile: Haiti. 2018.

47. Devlin K, Egan KF, Pandit-Rajani T. Community Health Systems Catalog Country Profile : Haiti. Arlington, VA; 2016.

48. Republic of Kenya. Constitution of Kenya. 2010.

49. Republic of Kenya Ministry of Health. Strategy for Community Health 2014-2019: Transforming health: Accelerating the attainment of health goals. 2014.

50. Republic of Uganda Ministry of Health. National Village Health Teams (VHT) assessment in Uganda. 2015.

51. Botha W, Donnolley N, Shanahan M, Chambers GM. Assessment of the societal and individual preferences for fertility treatment in Australia: study protocol for stated preference discrete choice experiments. BMJ Open. 2018;8(2).

52. DeShazo J, Fermo G. Designing choice sets for stated preference methods: the effects of complexity on choice consistency. J Env Econ Manag. 2002;44.

53. Sawtooth Software. CBC vs.ACBC: Comparing Results with Real Product Selection. 2007. p. 8.

54. Waylon B, Samuel S, Mitchell S, Clarice L, Jeffrey B, Anderson Richard. Open Data Kit 2.0: A Services-Based Application Framework for Disconnected Data Management. 2017.

55. de Bekker-Grob EW, Donkers B, Jonker MF, Stolk EA. Sample Size Requirements for Discrete-Choice Experiments in Healthcare: a Practical Guide. Patient - Patient-Centered Outcomes Res. 2015 Oct;8(5):373–84.

56. Orme B. Sample Size Issues for Conjoint Analysis. Sequim Sawtooth Softw Tech Pap. 1998;

57. Johnson R, Orme B. Getting the most from CBC. Sequim Sawtooth Softw Res Pap Ser Sawtooth Softw. 2003;

58. STATACorp. State statistical software. College Station, TX: STATACorp LLC; 2019.

Authors' contributions

SA conceptualized the study and led the writing of the first draft of the protocol. UA assisted with relevant background search and writing. CEW designed the overall frontline health worker study, TA, RK, AC, SH adapted the protocol for Kenya, Uganda, Haiti and Bangladesh, respectively. MO developed the statistical approach for the study. All authors supported the development of the research design and reviewed the final manuscript.

Funding statement

This study is funded by the Bill & Melinda Gates Foundation. The funding body has no role in the study design, data collection, analysis, or interpretation of data.

Conflict of interest's statement

For peer review only

The authors declare no conflict of interests

Data statement

Data can be made available on request after the initial analysis is completed and disseminated.

For peer review only
